# Effect of Ghrelin on Aldolase Gene Expression in the Heart of Chronic Hypoxic Rat

**DOI:** 10.5812/ijem.3914

**Published:** 2012-06-30

**Authors:** Mohammad Reza Aliparasti, Mohammad Reza Alipour, Shohreh Almasi, Hadi Feizi

**Affiliations:** 1 Immunology Research Center, Tabriz University of Medical Sciences, Tabriz, IR Iran; 2 Department of Immunology, Tabriz University of Medical Sciences, Tabriz, IR Iran; 3 Tuberculosis and Lung Research Center, Tabriz University of Medical Sciences, Tabriz, IR Iran; 4 Department of Physiology, Hormozgan University of Medical Sciences, Hormozgan, IR Iran

**Keywords:** Ghrelin, Cell Hypoxia, Heart, Glycolytic enzymes, Rat

## Abstract

**Background:**

Chronic hypoxia causes apoptosis of cardiac myocytes, however, energy production by anaerobic glycolysis protects myocardium against hypoxia injuries. Aldolase A is a well-characterised key enzyme of the glycolysis pathway. Ghrelin, a 28-amino-acid peptide, synthesizes in the stomach and has protective roles in cardiovascular systems and also affects metabolic pathways.

**Objectives:**

Therefore, the aim of this study was to evaluate the effect of ghrelin on aldolase A gene expression after chronic hypoxia in the rat hearts.

**Materials and Methods:**

Twenty four adult male wistar rats were randomly divided into three groups. Hypoxic rats with saline or ghrelin treatment were placed in a normobaric hypoxic chamber (O2 11 %), for two weeks. Controls remained in room air. Aldolase A gene expression was measured by Real-Time RT-PCR.

**Results:**

the transcriptiom rate of Aldolase A in hypoxic animals did not change significantly compared to negative control ones. During chronic hypoxia, ghrelin treatment increased the amount of heart Aldolase A gene expression compared to negative controls (P = 0.029). Hypoxic animals that were treated with ghrelin were significantly more polycythemic than the controls and even hypoxic with saline treated rats (P < 0.001).

**Conclusions:**

It seems that ghrelin interferes in the cardiac metabolism through upregulation of glycolytic enzymes. In other words, it may protect heart from possible hypoxia induced damages.

## 1. Background

Prolonged hypoxia causes cardiac myocytes apoptosis (programmed cell death) leading to myocardial dysfunction (1, 2). Enhanced energy production via anaerobic glycolysis is the major mechanisms promoting myocardial survival during hypoxia (3-7). Moreover, Malhotra et al. demonstrated that glycolysis of extracellular glucose, protects cardiac myocytes from hypoxic injury and subsequent apoptosis (8). One well-characterised key enzyme of the glycolytic pathway is aldolase (9). In higher vertebrates, 3 tissue-specific isoenzymes (A, B, and C type) are identified which aldolase A is found in muscle tissue (10). Furthermore, hypoxia response elements have been characterized in aldolase A (11, 12).

Ghrelin, a 28-amino-acid peptide, discovered in 1999 as an endogenous ligand of the growth hormone secretagogue receptor, synthesizes by the endocrine cells of the gastric mucosa and its receptor has been found in heart (13, 14). This peptide has direct beneficial effects on several aspects of heart function and protects myocytes from different injuries (15, 16). Ghrelin, can also attenuate cardiac dysfunction and energy metabolic disturbance in chronic heart failure (17). It has been revealed that ghrelin affects cardiac metabolism via AMP-activated Protein Kinase (18).

## 2. Objectives

Based on this background, the aim of this study was to evaluate the effect of ghrelin on aldolase A gene expression, in the heart of chronic hypoxic rats.

## 3. Materials and Methods

### 3.1. Animals and Chronic Hypoxia Model Design

All experiments on animal subjects were conducted in accordance with the highest ethical standards of the Medicine faculty, Tabriz University of Medical Sciences, Iran. Male adult wistar rats (200-250gr) were housed in cages in a temperature and light-controlled environment and provided with food and water ad libitum. Animals were randomly divided into 3 groups of 8 rats, including control (C), hypoxia with saline (H+S), and hypoxia with ghrelin (H+G). In hypoxic groups (H+S and H+G), hypoxia was induced by Environmental Chamber System GO2Altitude (Biomedtech Australia Pty. Ltd), which generates hypoxic air without the need for a gas cylinder. H+S and H+G animals were placed in a ventilated chamber inflated by hypoxic air (O_2_ 11 %), simulated to 5150 m above sea level. An O_2_ sensor and controller was embedded in the chamber wall to monitor the O_2_ concentration. Animals were kept in the chamber for two weeks except for 20 min/day to clean the cages and perform daily injections (19).

### 3.2. Drug Administration

Rats received a subcutaneous injection of either saline (0.1 ml) or ghrelin (150 µg/kg/day in 0.1 ml) (19), and were then placed into the hypoxic chamber. H+S and H+G rats continuously received daily injections of either saline or ghrelin during the 2 weeks. Ghrelin was obtained from the Tocris Bioscience Co. (Bristol, UK), and administered dissolved in saline as the vehicle.

### 3.3. RNA Extraction and First-Strand cDNA Synthesis

Total cellular RNA was extracted from rat heart tissue using Trizol Reagent(Invitrogen, USA) according to the manufacturer’s instruction,and the isolates were treated with RNase-free DNase to remove any residual genomic DNA. Single stranded cDNAs were synthesized by incubating total RNA (1 µg) with RevertAid H Minus M-MuL V Reverse transcriptase (200 U), oligo-(dT)_18_ primer (5 µM), Random Hexamer Primer (5 µM), dNTPs (1 mM), and RiboLock RNase-inhibitor (20 U), for 5 min at 25°C followed by 60 min incubation at 42°C in a final volume of 20 µl. Reaction was terminated by heating at 70°C for 5 min.

### 3.4. Real-Time Relative Quantitative RT-PCR

Quantitative Real Time PCR was performed using the Corbett Life Science (Rotor-Gene 6000) System, 2 µL of a 3-fold diluted cDNA were added in each PCR reaction with a final volume of 20 µL. Each PCR reaction contained 5 pM of primers and 1 × FastStart SYBR Green Master (Roche). primer Sequences are shown in Table 1. PCR amplification performed by three repeated cycles of temperature dependent steps : 1) denaturation of cDNA (1 cycle: 95°C for 10 min); 2) amplification (40 cycles: 95°C for 15 sec, 57°C for 30 sec 60°C for 34 sec); 3) melting curve analysis (1 cycle: 60 to 95°C with temperature transition rate 1°C/sec). A mixture of serially diluted cDNA of all samples were used to generate standard curves. β-actin (Actb) mRNA expression levels were applied to calculate relative expression levels. All data are presented as the ratio of the target geneto Actb. The relative quantification was calculated by 2(^−ΔCt^) : Expression of target genes/ β-actin = (1+E) ^-Ct^ target gene/ (1+E) ^-Ct^ β-actin. The PCR reaction specificity was verified by generation of a melting curve analysis followed by gel electrophoresis, visualized by ethidium bromide staining (19).

**Table 1:** Sequences of Oligonucleotide Primers

	Forward Primer	Reverse Primer	Product Size (bp)
Aldolase A	ATGCCCCACCCATACCCAGCACT	AGCAGCAGTTGGCGGTAGAAGCG	191
β-actin	TCCTCCTGAGCGCAAGTACTCT	GCTCAGTAACAGTCCGCCTAGAA	153

### 3.5. Statistical Analysis

Expression of Aldolase A was obtained through the Corbett Rotor-Gene 6000 and expressed as Ct (cycle threshold), ΔCt (Ct of target gene – Ct of House keeping gene). The collected data were analyzed using statistical SPSS software, version 16. Variables were reported as means with standard deviations. Data were analyzed by one-way ANOVA to evaluate differences between groups. according to equality of variancespost hoc analyses were performed using the Tukey tests For multiple comparisons which statistical significance was reached.

## 4. Results

### 4.1. Hematocrit Measurement

**Figure 1:**
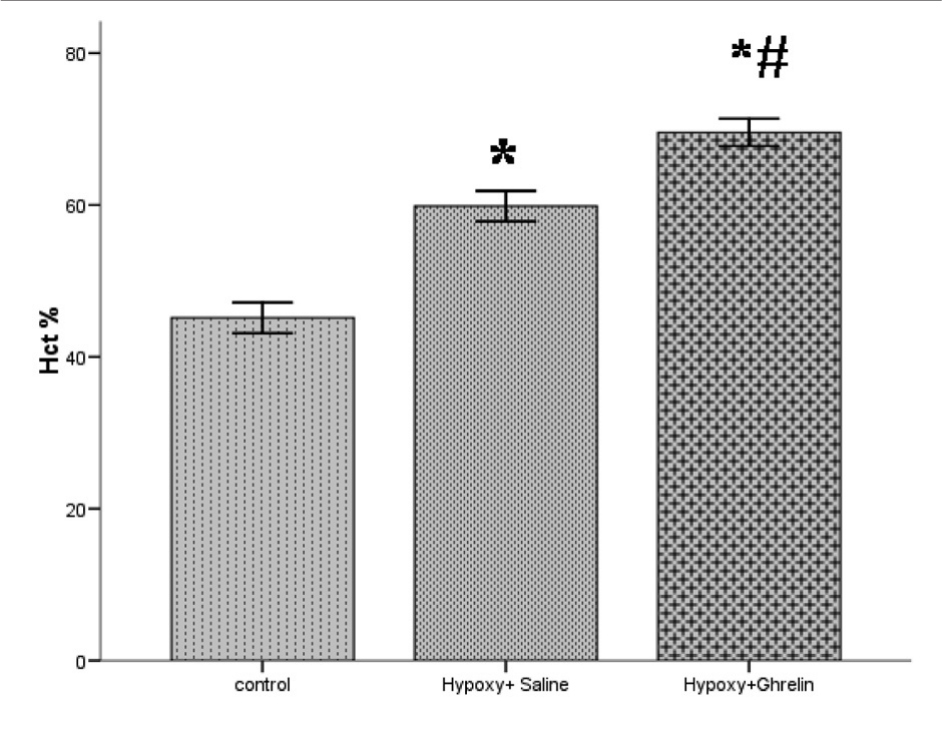
Average Hematocrit After Two Weeks in Control, Hypoxic With Saline, And Hypoxic With Ghrelin Groups. Data are reported as mean ± SEM.*(p < 0.001) significant difference compared with control subjects and # (p < 0.001) significant difference compared with H+S. Ghrelin was injected subcutaneously (150 μg/kg/day in 0.1 ml).

After two weeks of treatment, the average hematocrit of C, H+S and H+G groups were 45.14 % ± 1.01, 59.10 % ± 1.37 and 69.57 % ± 0.89 respectively in which a significant polycythemia occurred in H+S and H+G animals compared with the C group ( P < 0 .0001) (Figure 1).

### 4.2. Effect of Hypoxia on Aldolase Gene Expression

**Figure 2:**
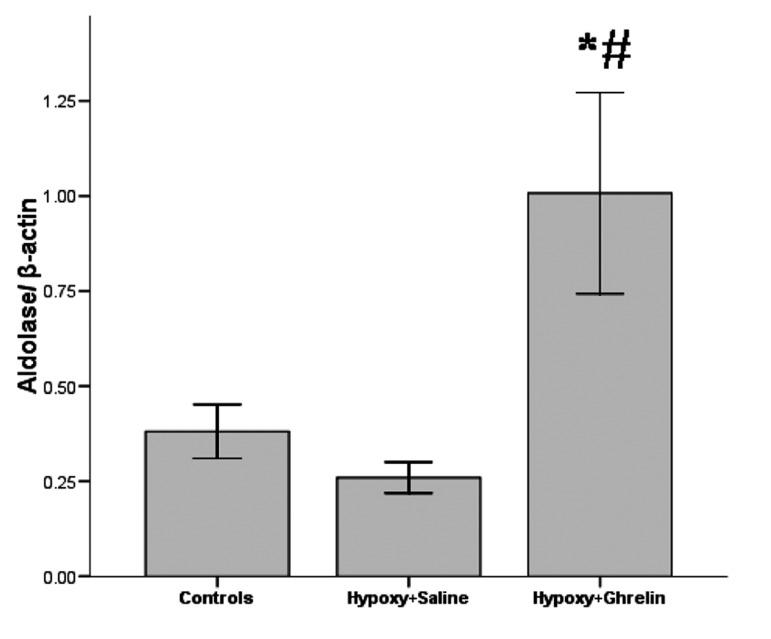
Relative Quantitative RT-PCR of Aldolase A to β-actin (n = 8). Data are presented as mean ± SEM. * significandifference from normoxia (P < 0.05). ≠ significant difference between chronic hypoxic rats treated with saline v.s ghrelin (P < 0.05).

After 2-weeks of hypoxia, Aldolase A transcripts of hypoxic animals did not change significantly compared to control animals (P = 0.845) (Figure 2).

### 4.3. Effect of Ghrelin on Aldolase Gene Expression During Hypoxia

During chronic hypoxia, ghrelin treatment increased the amount of heart Aldolase A gene expression compared to normal controls (P = 0.029) (Figure 2). Furthermore, data analysis showed that expression of Aldolase A in the H+G heart animals was increased by 3.87- fold compared with hypoxic group (P = 0.009).

## 5. Discussion

To validate our hypoxic model, it was necessary to measurehe matocrit level of different groups. Interestingly, severe polycithemia has been observed among hypoxic animals treated with ghrelin. In the present study, the mechanism of ghrelin reaction was not completely understood; however, ghrelin effect on hematopoisis systems including erythropoietin in the kidney and stress erythropoiesis in spleen is a subject for further studies. It must be noted that ghrelin receptors are presented in the mentioned organs. Although it seems that ghrelin leads to an increase in blood viscosity, but is supported by its well-known vasodilating effect (20-22), the adverse outcomes will be neutralized and slight changes will be added to total peripheral resistance (TPR) and cardiac performance.

According to the result of this study, hypoxic condition did not cause significant change in gene expression of glycolytic enzyme aldolase A in the heart. This finding is in conformity with those reported by Martinez et al. (23). Based on the result of this study, ghrelin treatment increased the expression of Aldolase A. Considering the fact that the cells must be able to maintain glycolysis at a level that is sufficient to sustain ATP under hypoxia, this finding introduces a positive effect of ghrelin. However, the mechanism of this effect of ghrelin could be under scrutiny. To date, many investigating groups have found evidences that ghrelin exerts potent cardiovascular protections on various cardiovascular diseases such as heart failure, myocardial infarction and pulmonary hypertension (15-17). In 2005, Kola and coworkers reported that ghrelin stimulates AMPK activity in the heart (24). Since the AMPK has a prominent role in energy sensing and metabolism (25, 26), the present findings provide important evidence of interaction between ghrelin and metabolism regulation in hypoxic conditions, probably through AMPK. As reported by Xu et al., it is also remarkable that exogenous administration of ghrelin could be a new therapeutic approach to the treatment of severe CHF because it can attenuate the myocardial metabolic disorders (27). Another fact is that chronic peripheral ghrelin administration to lean rats up-regulates AKT activation in the muscle (28). It has been reported that Ghrelin inhibits apoptosis in cultured cardiomyocytes and endothelial cells through activation of ERK1/2 and AKT serine kinase (29). Given the proposed role of AKT in glucose metabolism (30), this is an additional mechanism that ghrelin possibly affects this glycotic enzyme activity. Despite the beneficiary effect of promoting glycolysis with the purpose of continuous supply of glucose in hypoxic condition, the cells must be able to clear the excess acid produced by anaerobic glycolysis. As follows, other researchers believe that glycolysis could aggravates the condition by producing lactic acid (31, 32). However, this uncomplimentary effect is repeatedly marked in cardiac myocytes of failing hearts, but not those of normal hearts in which adaptations to hypoxia activate more efficiently (33).

In conclusion, ghrelin probably supports heart metabolic needs in hypoxic conditions by induction of glycolysis enzymes but the molecular mechanisms should be elucidated.

## References

[1] Jung F, Weiland U, Johns RA, Ihling C, Dimmeler S (2001). Chronic hypoxia induces apoptosis in cardiac myocytes: a possible role for Bcl-2-like proteins.. Biochem Biophys Res Commun..

[2] Tanaka M, Ito H, Adachi S, Akimoto H, Nishikawa T, Kasajima T (1994). Hypoxia induces apoptosis with enhanced expression of Fas antigen messenger RNA in cultured neonatal rat cardiomyocytes.. Circ Res..

[3] Bekheit S, Isber N, Jani H, Butrous G, Boutjdir M, el-Sherif N (1993). Reduction of ischemia-induced electrophysiologic abnormalities by glucose-insulin infusion.. J Am Coll Cardiol..

[4] Eberli FR, Weinberg EO, Grice WN, Horowitz GL, Apstein CS (1991). Protective effect of increased glycolytic substrate against systolic and diastolic dysfunction and increased coronary resistance from prolonged global underperfusion and reperfusion in isolated rabbit hearts perfused with erythrocyte suspensions.. Circ Res..

[5] Kim JW, Tchernyshyov I, Semenza GL, Dang CV (2006). HIF-1-mediated expression of pyruvate dehydrogenase kinase: a metabolic switch required for cellular adaptation to hypoxia.. Cell Metab..

[6] Opie LH (1995). Glucose and the metabolism of ischaemic myocardium.. Lancet..

[7] Owen P, Dennis S, Opie LH (1990). Glucose flux rate regulates onset of ischemic contracture in globally underperfused rat hearts.. Circ Res..

[8] Malhotra R, Brosius FC, 3rd (1999). Glucose uptake and glycolysis reduce hypoxia-induced apoptosis in cultured neonatal rat cardiac myocytes.. J Biol Chem..

[9] Penhoet E, Kochman M, Valentine R, Rutter WJ (1967). The subunit structure of mammalian fructose diphosphate aldolase.. Biochemistry..

[10] Penhoet E, Rajkumar T, Rutter WJ (1966). Multiple forms of fructose diphosphate aldolase in mammalian tissues.. Proc Natl Acad Sci U S A..

[11] Semenza GL, Jiang BH, Leung SW, Passantino R, Concordet JP, Maire P (1996). Hypoxia response elements in the aldolase A, enolase 1, and lactate dehydrogenase A gene promoters contain essential binding sites for hypoxia-inducible factor 1.. J Biol Chem..

[12] Semenza GL, Roth PH, Fang HM, Wang GL (1994). Transcriptional regulation of genes encoding glycolytic enzymes by hypoxia-inducible factor 1.. J Biol Chem..

[13] Davenport AP, Bonner TI, Foord SM, Harmar AJ, Neubig RR, Pin JP (2005). International Union of Pharmacology. LVI. Ghrelin receptor nomenclature, distribution, and function.. Pharmacol Rev..

[14] Kojima M, Hosoda H, Date Y, Nakazato M, Matsuo H, Kangawa K (1999). Ghrelin is a growth-hormone-releasing acylated peptide from stomach.. Nature..

[15] Garcia EA, Korbonits M (2006). Ghrelin and cardiovascular health.. Curr Opin Pharmacol..

[16] Kishimoto I, Tokudome T, Schwenke DO, Takeshi S, Hosoda H, Nagaya N (2009). Therapeutic potential of ghrelin in cardiac diseases.. Expert Review of Endocrinology and Metabolism..

[17] Nagaya N, Uematsu M, Kojima M, Ikeda Y, Yoshihara F, Shimizu W (2001). Chronic administration of ghrelin improves left ventricular dysfunction and attenuates development of cardiac cachexia in rats with heart failure.. Circulation..

[18] van Thuijl H, Kola B, Korbonits M (2008). Appetite and metabolic effects of ghrelin and cannabinoids: involvement of AMP-activated protein kinase.. Vitam Horm..

[19] Alipour MR, Aliparasti MR, Keyhanmanesh R, Almasi S, Halimi M, Ansarin K (2011). Effect of ghrelin on protein kinase C-epsilon and protein kinase C-delta gene expression in the pulmonary arterial smooth muscles of chronic hypoxic rats.. J Endocrinol Invest..

[20] Nagaya N, Kojima M, Uematsu M, Yamagishi M, Hosoda H, Oya H (2001). Hemodynamic and hormonal effects of human ghrelin in healthy volunteers.. Am J Physiol Regul Integr Comp Physiol..

[21] Okumura H, Nagaya N, Enomoto M, Nakagawa E, Oya H, Kangawa K (2002). Vasodilatory effect of ghrelin, an endogenous peptide from the stomach.. J Cardiovasc Pharmacol..

[22] Wiley KE, Davenport AP (2002). Comparison of vasodilators in human internal mammary artery: ghrelin is a potent physiological antagonist of endothelin-1.. Br J Pharmacol..

[23] Martinez ML, Landry C, Boehm R, Manning S, Cheek AO, Rees BB (2006). Effects of long-term hypoxia on enzymes of carbohydrate metabolism in the Gulf killifish, Fundulus grandis.. J Exp Biol..

[24] Kola B, Hubina E, Tucci SA, Kirkham TC, Garcia EA, Mitchell SE (2005). Cannabinoids and ghrelin have both central and peripheral metabolic and cardiac effects via AMP-activated protein kinase.. J Biol Chem..

[25] Marsin AS, Bouzin C, Bertrand L, Hue L (2002). The stimulation of glycolysis by hypoxia in activated monocytes is mediated by AMP-activated protein kinase and inducible 6-phosphofructo-2-kinase.. J Biol Chem..

[26] Russell RR, 3rd, Li J, Coven DL, Pypaert M, Zechner C, Palmeri M (2004). AMP-activated protein kinase mediates ischemic glucose uptake and prevents postischemic cardiac dysfunction, apoptosis, and injury.. J Clin Invest..

[27] Xu JP, Wang HX, Wang W, Zhang LK, Tang CS (2010). Ghrelin improves disturbed myocardial energy metabolism in rats with heart failure induced by isoproterenol.. J Pept Sci..

[28] Barazzoni R, Zanetti M, Cattin MR, Visintin L, Vinci P, Cattin L (2007). Ghrelin enhances in vivo skeletal muscle but not liver AKT signaling in rats.. Obesity (Silver Spring)..

[29] Baldanzi G, Filigheddu N, Cutrupi S, Catapano F, Bonissoni S, Fubini A (2002). Ghrelin and des-acyl ghrelin inhibit cell death in cardiomyocytes and endothelial cells through ERK1/2 and PI 3-kinase/ AKT.. J Cell Biol..

[30] Cross DA, Alessi DR, Cohen P, Andjelkovich M, Hemmings BA (1995). Inhibition of glycogen synthase kinase-3 by insulin mediated by protein kinase B.. Nature..

[31] Kubasiak LA, Hernandez OM, Bishopric NH, Webster KA (2002). Hypoxia and acidosis activate cardiac myocyte death through the Bcl-2 family protein BNIP3.. Proc Natl Acad Sci U S A..

[32] Luo F, Liu X, Yan N, Li S, Cao G, Cheng Q (2006). Hypoxia-inducible transcription factor-1alpha promotes hypoxia-induced A549 apoptosis via a mechanism that involves the glycolysis pathway.. BMC Cancer..

[33] Todor A, Sharov VG, Tanhehco EJ, Silverman N, Bernabei A, Sabbah HN (2002). Hypoxia-induced cleavage of caspase-3 and DFF45/ICAD in human failed cardiomyocytes.. Am J Physiol Heart Circ Physiol..

